# Deep learning radiomics model of epicardial adipose tissue for predicting postoperative atrial fibrillation after lung lobectomy in lung cancer patients

**DOI:** 10.3389/fonc.2025.1623248

**Published:** 2025-10-13

**Authors:** Zhan Liu, Chong Zheng, Zongxiao Jia, Chengwei Zhao, Xiangyu Liu, Weipeng Shao, Feng Chen, Hui Zhu, Hongbo Guo

**Affiliations:** ^1^ Department of Lung Surgical Ward II, Shandong Cancer Hospital and Institute, Shandong First Medical University and Shandong Academy of Medical Sciences, Jinan, Shandong, China; ^2^ Department of Radiology, Shandong Cancer Hospital and Institute, Shandong First Medical University and Shandong Academy of Medical Sciences, Jinan, Shandong, China; ^3^ Department of Thoracic Surgical, Feicheng Hospital Affiliated to Shandong First Medical University, Taian, Shandong, China; ^4^ Department of Radiation Oncology, Shandong Cancer Hospital and Institute, Shandong First Medical University and Shandong Academy of Medical Sciences, Jinan, Shandong, China

**Keywords:** postoperative atrial fibrillation, deep learning radiomics, epicardial adipose tissue, lung lobectomy, lung cancer

## Abstract

**Objective:**

To develop and validate a deep learning (DL) radiomics model based on epicardial adipose tissue (EAT) for identifying high-risk lung cancer patients with postoperative atrial fibrillation after lung lobectomy.

**Methods:**

A total of 1,008 patients from two centers were included. Handcrafted and DL radiomics features were extracted from the preoperative contrast-enhanced chest CT images of EAT. Clinical features and handcrafted and DL radiomics signatures were integrated to construct predictive models using the logistic regression algorithm as the baseline model. Twenty DL radiomics models were constructed through various combinations of machine learning algorithms and resampling techniques. The *post hoc* Nemenyi test was employed to compare the predictive performance in terms of the area under the receiver operating characteristic curve (AUC), G-mean, and F-measure.

**Results:**

Advanced age and male sex were identified as independent risk factors for POAF. The DL radiomics model, integrating clinical features, handcrafted radiomics signature, and DL radiomics signature, outperformed the clinical model, achieving AUC values of 0.890 (95% CI: 0.816–0.963), 0.876 (95% CI: 0.755–0.997), and 0.803 (95% CI: 0.651–0.955) in the training, testing, and validation cohorts, respectively. The results of the *post hoc* Nemenyi tests indicated that neither machine learning algorithms nor resampling techniques significantly improved model performance, as measured by the AUC, G-mean, or F-measure.

**Conclusion:**

The DL radiomics model based on preoperative EAT images effectively identifies high-risk lung cancer patients with POAF following lung lobectomy and offers a novel tool for risk stratification.

## 1 Introduction

Lung lobectomy is the most common operation performed for lung cancer patients. Postoperative atrial fibrillation (POAF) is a common complication following lung lobectomy, with incidence rates ranging from 5.2% to 17.6% (1–4). Although POAF is often considered transient and self-limiting, it is significantly associated with prolonged hospital stays, increased risk of stroke, and higher mortality rates (5–7). Studies have shown that perioperative strategies, such as the administration of magnesium sulfate, preferential use of vasopressors over inotropes, avoidance of red cell transfusion, and video-assisted thoracoscopic surgery, may reduce POAF incidence (3, 8). Therefore, accurate preoperative risk stratification and tailored perioperative management are essential for improving outcomes and quality of life in high-risk lung cancer patients.

Several clinical characteristics, including advanced age, male sex, and procedural invasiveness, have been identified as key predictors of POAF (7, 9). Although these risk factors have been incorporated into predictive models, their performance remains suboptimal, with area under the receiver operating characteristic curve (AUC) values typically below 0.80 (3, 4, 10). Consequently, improving the identification of high-risk patients with POAF is warranted. Epicardial adipose tissue (EAT), a unique fat depot located between the myocardium and the visceral layer of the epicardium, has been suggested to play a role in the development and progression of atrial fibrillation (11). West et al. demonstrated that EAT volume could predict both in-hospital and long-term post-cardiac surgery atrial fibrillation (12).

Radiomics, which extracts high-throughput quantitative features from medical images, can provide disease-related information beyond traditional quantitative features such as volume and density (13, 14). Several studies have highlighted the effectiveness of EAT radiomics models in predicting POAF (15–17). The integration of deep learning (DL) radiomics features, particularly 3D DL features, enhances radiomics by capturing intricate structural patterns, thereby improving predictive model performance (18, 19). Therefore, integrating radiomics and DL analysis of EAT on preoperative CT images may offer a novel and robust approach for predicting POAF following lung lobectomy.

In this study, we aimed to develop and validate a DL radiomics model based on EAT to identify high-risk lung cancer patients for POAF following lung lobectomy. Additionally, we systematically evaluated the impact of various machine learning algorithms and resampling techniques on model performance.

## 2 Materials and methods

### 2.1 Study cohorts

This study was approved by the Ethics Board of Shandong Cancer Hospital and Institute, Shandong First Medical University and Shandong Academy of Medical Sciences (SDTHEC 202411027), and Feicheng Hospital Affiliated to Shandong First Medical University (2024039). Informed consent was waived due to the retrospective design of the study.

Between 1 May 2023 and 31 October 2023, 890 consecutive lung cancer patients who underwent lung lobectomy at, Shandong First Medical University and Shandong Academy of Medical Sciences (center 1) were enrolled. Additionally, 118 consecutive patients who underwent lung lobectomy for lung cancer at Feicheng Hospital Affiliated to Shandong First Medical University (center 2) between 1 May 2021 and 31 October 2023 were also included. The inclusion criteria were as follows: 1) underwent lung lobectomy for lung cancer and 2) preoperative contrast-enhanced chest CT performed within 1 week before surgery. The exclusion criteria were as follows: 1) prior history of atrial fibrillation or atrial flutter, 2) prior history of open heart surgery, 3) missing or incomplete clinical data, and 4) inadequate CT image quality for analysis. The workflow of this study is shown in **Supplementary Figure S1**.

Preoperative demographic data, comorbidities, electrocardiogram findings, and hematologic examination results were collected from the electronic medical record system. All patients underwent continuous telemetry monitoring for at least 48 to 72 h postoperatively, with extended monitoring as clinically indicated. POAF was defined as new-onset atrial fibrillation lasting >5 min, detected by continuous telemetry or 12-lead electrocardiogram following lung lobectomy.

### 2.2 CT examination and image preprocessing

All patients underwent contrast-enhanced chest CT examination using a multidetector CT system within 1 week prior to surgery. The scanning parameters are shown in **Supplementary Table S1**. Iodinated contrast agent (300 mg/mL) at a dose of 1.5 mL/kg body weight was injected rapidly at a flow rate of 2 mL/s through the patient’s elbow vein using a high-pressure syringe. Arterial phase CT images were retrieved from the Picture Archiving and Communication Systems for further evaluation. Normalization was performed on all images based on the mean and variance.

EAT segmentation was automatically performed using the TIMESlice software (version 4.19.0, https://slice-doc.netlify.app/) (20). First, the pericardium was delineated from the diaphragm to the pulmonary artery bifurcations. Then, a segmentation algorithm based on a Hounsfield unit (HU) threshold (between −190 and −30 HU) was used to identify EAT. After the automatic segmentation of EAT was completed, two experienced radiologists reviewed and adjusted the volume of interest (VOI). EAT images are shown in **Supplementary Figure S2**.

### 2.3 Radiomics feature extraction

The Python software (version 3.9.13, https://www.python.org/) and the PyRadiomics package were used to extract handcrafted radiomics features from the VOIs. A fixed bin width of 25 was set for image discretization. Bicubic spline interpolation was used to resample the original images to a voxel size of 1 mm × 1 mm × 1 mm. Finally, a total of 1,130 handcrafted radiomics features were extracted from each VOI of the original images and their corresponding filtered, transformed images. The pretrained 3D ResNet-18 model provided by torchvision, which was originally trained on the Kinetics-400 dataset, was employed to extract 512 DL features. To adapt the model to medical imaging data, the input layer was modified to accept single-channel (grayscale) input instead of the original three-channel (RGB) input. Additionally, the final fully connected layer was removed to adapt the model for use as a feature extractor.

### 2.4 Statistical analyses

#### 2.4.1 Cohort splitting

Patients from center 1 were grouped by stratified random sampling based on the clinical outcome (with or without POAF) in a ratio of 7:3, with 623 and 267 patients in the training and testing cohorts, respectively. The validation cohort comprised 118 patients from center 2. Different resampling techniques, including random oversampling (ROS), random oversampling examples (ROSE), synthetic minority oversampling technique (SMOTE), and Borderline-SMOTE (bSMOTE), were applied to the training cohort to address the class imbalance distribution between patients with and without POAF.

#### 2.4.2 Radiomics signature construction

Radiomics feature selection and signature construction were performed in the training cohort. First, the handcrafted and DL radiomics features were standardized using *z*-score normalization to eliminate differences introduced by value scales between radiomics features. The radiomics features in the testing and validation cohorts were normalized based on the mean value and standard deviation derived from the training cohort. The Spearman or Pearson correlation coefficients for each pair of radiomics features were calculated, and redundant features with a correlation coefficient greater than 0.9 were removed. The max-relevance and min-redundancy (mRMR) algorithm was implemented to rank the importance of the radiomics features and select the top 30 most significant features for subsequent analysis. Subsequently, the least absolute shrinkage and selection operator (LASSO) algorithm was applied to identify significant radiomics features with non-zero coefficients. The handcrafted and DL radiomics signatures were constructed using a linear combination of the final selected features and their corresponding coefficients.

#### 2.4.3 Predictive models construction

The differences in the clinical features between patients in different groups or cohorts were compared using the Student’s *t*-test or Mann–Whitney *U* test for continuous variables and the chi-squared test for categorical variables, as appropriate. Clinical features potentially associated with POAF (*P*<0.05) were then included in the multivariate logistic regression analysis to identify the independent risk factors.

The clinical model and combined models, including the clinical + handcrafted model, clinical + DL model, and clinical + handcrafted + DL model, were constructed using the logistic regression (LR) algorithm based on the selected clinical features and handcrafted and DL radiomics signatures in the training cohort. Furthermore, several machine learning algorithms, such as support vector machine (SVM), random forest (RF), and eXtreme Gradient Boosting (XGBoost), were also considered. The optimal hyperparameters of the classifiers were determined through a five-fold cross-validation method.

#### 2.4.4 Predictive models evaluation

The receiver operating characteristic (ROC) curves, AUC, sensitivity, specificity, accuracy, G-mean, and F-measure were used to assess the performance of the predictive models. The optimal classification threshold was determined using the Youden index (sensitivity + specificity − 1). The Delong test was used to compare the AUC values between the combined models and the clinical model. The net reclassification index (NRI) was also calculated to evaluate the incremental value of handcrafted radiomics signatures and DL radiomics signatures for POAF prediction. The *post hoc* Nemenyi test was adopted to compare the predictive performance of different combinations of resampling techniques and machine learning algorithms, and the results were visualized using critical difference (CD) plots.

The sample size was estimated using the “pmsampsize” package (21). Statistical analyses were conducted using R software (version 4.1.1, https://www.r-project.org/). A *P*-value <0.05 was considered statistically significant.

## 3 Results

### 3.1 Patient characteristics

A total of 1,008 lung cancer patients from one high-volume center (center 1, 890 patients) and one low-volume center (center 2, 118 patients) were included in this study, with 30 (3.4%) and 10 (8.5%) patients developing POAF, respectively. No significant differences were observed between patients in the training and testing cohorts (**Supplementary Table S2**). Compared to the training cohort, the validation cohort had a significantly higher proportion of POAF patients (8.5% vs. 3.4%, *P*=0.022) (**Supplementary Table S3**).

In the training cohort, age (*P*=0.020), sex (*P*=0.003), history of coronary heart disease (*P*=0.028), smoking history (*P*=0.021), lymphocyte count (*P*=0.022), and use of calcium channel blockers (*P*=0.046) were significantly different between patients with and without POAF (**Table 1**). The multivariate analysis showed that age (OR=1.079, 95% CI: 1.016–1.146, *P*=0.014) and male sex (OR=3.401, 95% CI: 1.125–10.286, *P*=0.030) were independent risk factors for POAF. Furthermore, we found that in the three cohorts, CHADS_2_ score, CHA_2_DS_2_-VASc score, and Passman score showed no significant differences between POAF and non-POAF patients (*P*<0.05). Clinical features between POAF and non-POAF patients in the testing and validation cohorts are described in **Supplementary Tables S4** and **S5**.

**Table 1:** Clinical features between patients with and without POAF in the training cohorts.

Features	Non-POAF *n*=602	POAF *n*=21	*P*-value
Sex			0.020
Female	285 (47.4)	4 (19.0)	
Male	317 (52.6)	17 (81.0)	
Age (years)	62 (56, 69)	68 (65, 72)	0.003
BMI (kg/m^2^)	24.2 (22.0, 26.7)	24.8 (21.7, 27.4)	
Hypertension	183 (30.4)	10 (47.6)	0.151
DM	67 (11.1)	2 (9.5)	1.000
CAD	49 (8.2)	5 (23.8)	0.028
CVD	42 (7.0)	4 (19.4)	0.061
PAD	15 (2.5)	1 (4.8)	0.426
Smoking history	210 (34.9)	13 (61.9)	0.021
Heart rate (bpm)	68 (61, 76)	71 (67, 85)	0.081
WBC (10^9^/L)	5.54 (4.51, 6.69)	5.76 (4.73, 7.01)	0.412
Neutrophil (10^9^/L)	3.24 (2.44, 4.15)	3.45 (2.58, 4.83)	0.210
Lymphocytes (10^9^/L)	1.65 (1.34, 1.98)	1.27 (1.16, 1.68)	0.022
Platelets (10^9^/L)	231 (195, 276)	221 (192, 285)	0.896
CCB	76 (12.6)	6 (28.6)	0.046
Metoprolol	25 (4.2)	1 (4.8)	0.598
Neoadjuvant therapy	80 (13.3)	6 (28.6)	0.056
CHADS_2_ score ≥2	83 (13.8)	5 (23.8)	0.200
CHA_2_DS_2_-VASc score ≥5	17 (2.8)	1 (4.8)	0.465
Passman score ≥4	196 (32.6)	10 (47.6)	0.228

Categorical variables shown with frequency and percentage; continuous variables shown with median and interquartile range.

POAF, postoperative atrial fibrillation; BMI, body mass index; DM, diabetes mellitus; CAD, coronary heart disease; CVD, cerebrovascular disease; PAD, peripheral arterial disease; bpm, beats per minute; WBC, white blood cell; CCB, calcium channel blocker.

We also adopted four different resampling techniques on the training cohort to balance the distribution between patients with and without POAF. The distribution of POAF and non-POAF patients before and after applying different resampling techniques is illustrated in **Supplementary Figure S3**.

### 3.2 Radiomics signature construction

**Figure 1:**
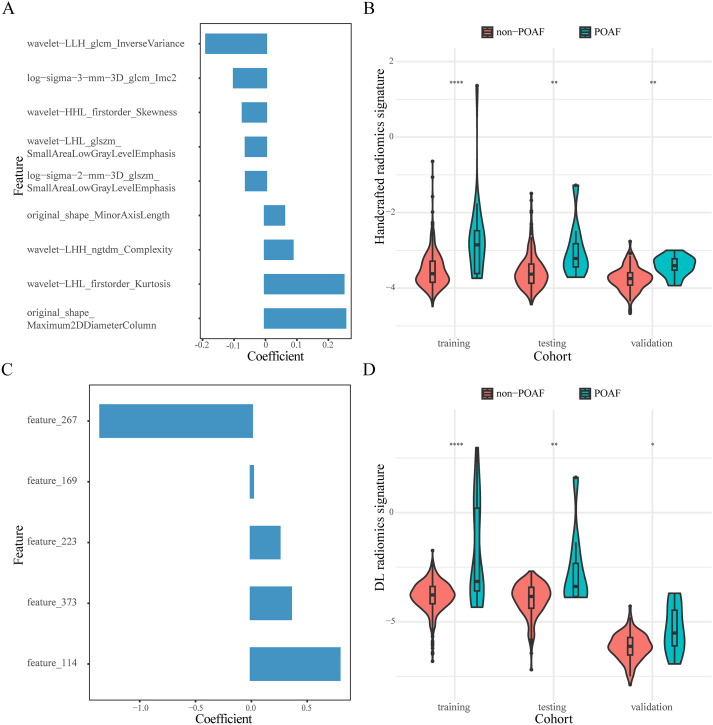
Composition features and distribution of constructed radiomics signatures. The selected handcrafted **(A)** and DL radiomics features **(C)**. Violin plots of the distribution of handcrafted **(B)** and DL radiomics signatures **(D)** in the three cohorts. *, P<0.05; **, P<0.01; ****, P<0.0001.

We selected nine handcrafted radiomics features and five DL radiomics features from the training cohort. The detailed information on the selected features and their coefficients is shown in **Figure 1**. In the original dataset, testing cohort, and validation cohort, the handcrafted and DL radiomics signatures were significantly elevated in patients with POAF (*P*<0.05; **Figure 1**). The distribution of constructed handcrafted and DL radiomics signatures between patients with and without POAF in the other resampling datasets is demonstrated in **Supplementary Figure S4**.

### 3.3 Model performance comparison

**Figure 2:**
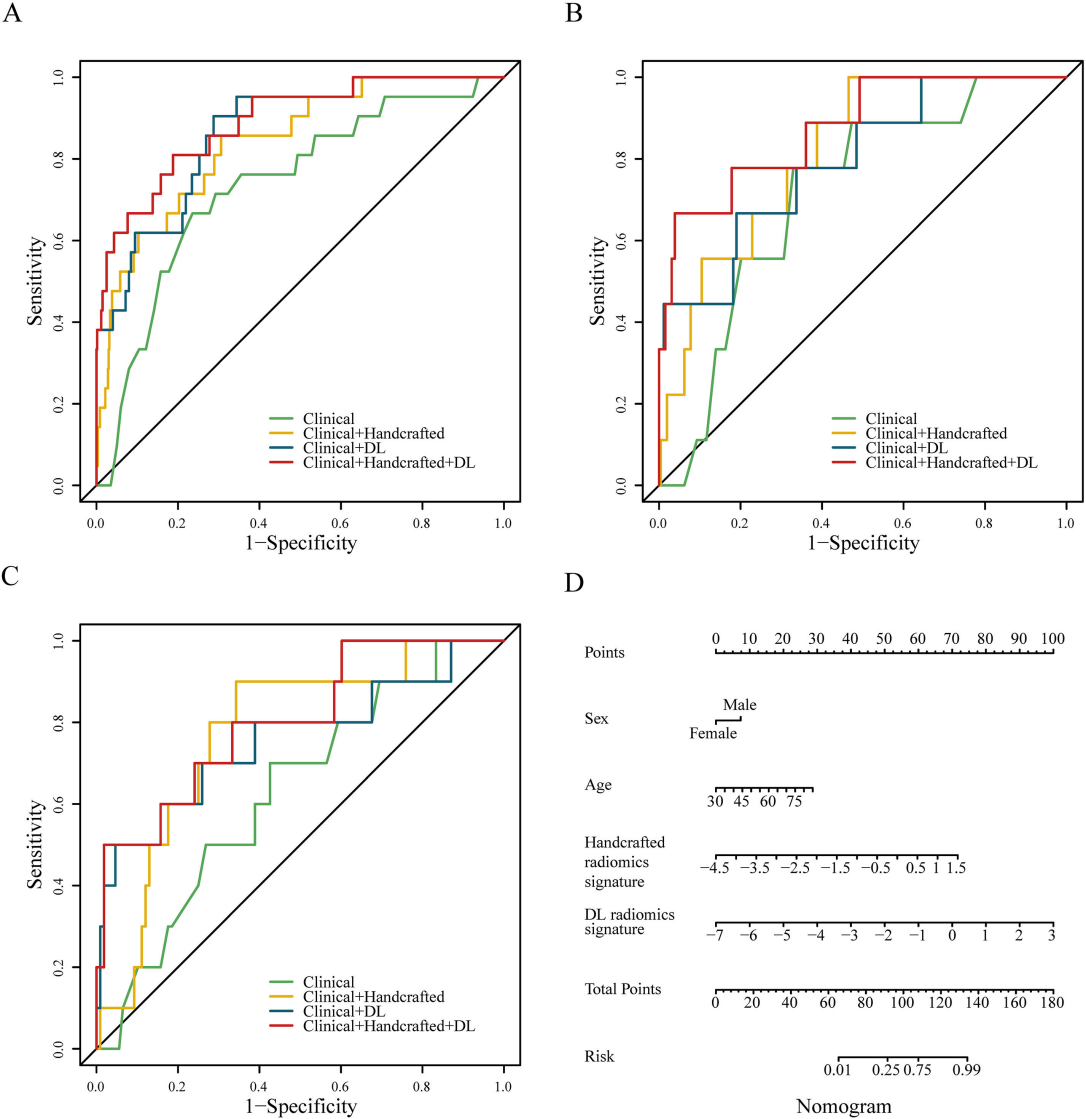
Receiver operating characteristic curves of four different predictive models constructed with no resampling technology and LR algorithm in the training **(A)**, testing **(B)**, and validation **(C)** cohorts. The nomogram of the clinical + handcrafted + DL model **(D)**.

We constructed four predictive models using the LR algorithm based on the independent clinical features and handcrafted and DL radiomics signatures. As shown in **Figure 2** and **Table 2**, the clinical + handcrafted + DL model, integrating clinical features and handcrafted and DL radiomics signatures, demonstrated superior predictive performance in the training, testing, and validation cohorts, with AUC values of 0.890, 0.876, and 0.803, respectively. Furthermore, the Delong test showed that the AUC of the clinical + handcrafted + DL model was significantly better than that of the clinical model in the training (*P*=0.016), testing (*P*=0.043), and validation (*P*=0.018) cohorts (**Table 2**). The NRI also indicated that the classification accuracy of POAF prediction improved significantly after integrating handcrafted and DL radiomics signatures compared to the clinical model, with *P*-values of 0.025, <0.001, and 0.004 in the three cohorts, respectively. Furthermore, the G-mean and F-measure of the clinical + handcrafted + DL model were higher than those of other models in all three cohorts (**Table 2**).

**Table 2:** Predictive performance of the models constructed with no resampling technology and LR algorithm.

Model	AUC	Sensitivity	Specificity	Accuracy	G-mean	F-measure
Training cohort						
Model 1	0.737 (0.628–0.847)	0.667	0.764	0.761	0.245	0.159
Model 2	0.841 (0.758–0.925)	0.857	0.694	0.700	0.276	0.161
Model 3	0.866 (0.794–0.937)	0.905	0.713	0.719	0.299	0.178
Model 4	0.890 (0.816–0.963)	0.810	0.812	0.812	0.326	0.226
Testing cohort						
Model 1	0.716 (0.570–0.862)	0.778	0.671	0.674	0.243	0.138
Model 2	0.815 (0.700–0.930)	1.000	0.535	0.551	0.265	0.131
Model 3	0.795 (0.637–0.952)	0.667	0.810	0.805	0.270	0.187
Model 4	0.876 (0.755–0.997)	0.667	0.961	0.951	0.500	0.480
Validation cohort						
Model 1	0.630 (0.458–0.802)	0.700	0.574	0.585	0.304	0.222
Model 2	0.773 (0.632–0.914)	0.900	0.657	0.678	0.420	0.322
Model 3	0.757 (0.561–0.952)	0.500	0.954	0.915	0.500	0.500
Model 4	0.803 (0.651–0.955)	0.500	0.981	0.941	0.597	0.588

Model 1, clinical model; model 2, clinical + handcrafted model; model 3, clinical + DL model; model 4, clinical + handcrafted + DL model; AUC, the area under the receiver operating characteristic curve; DL, deep learning.

**Figure 3:**
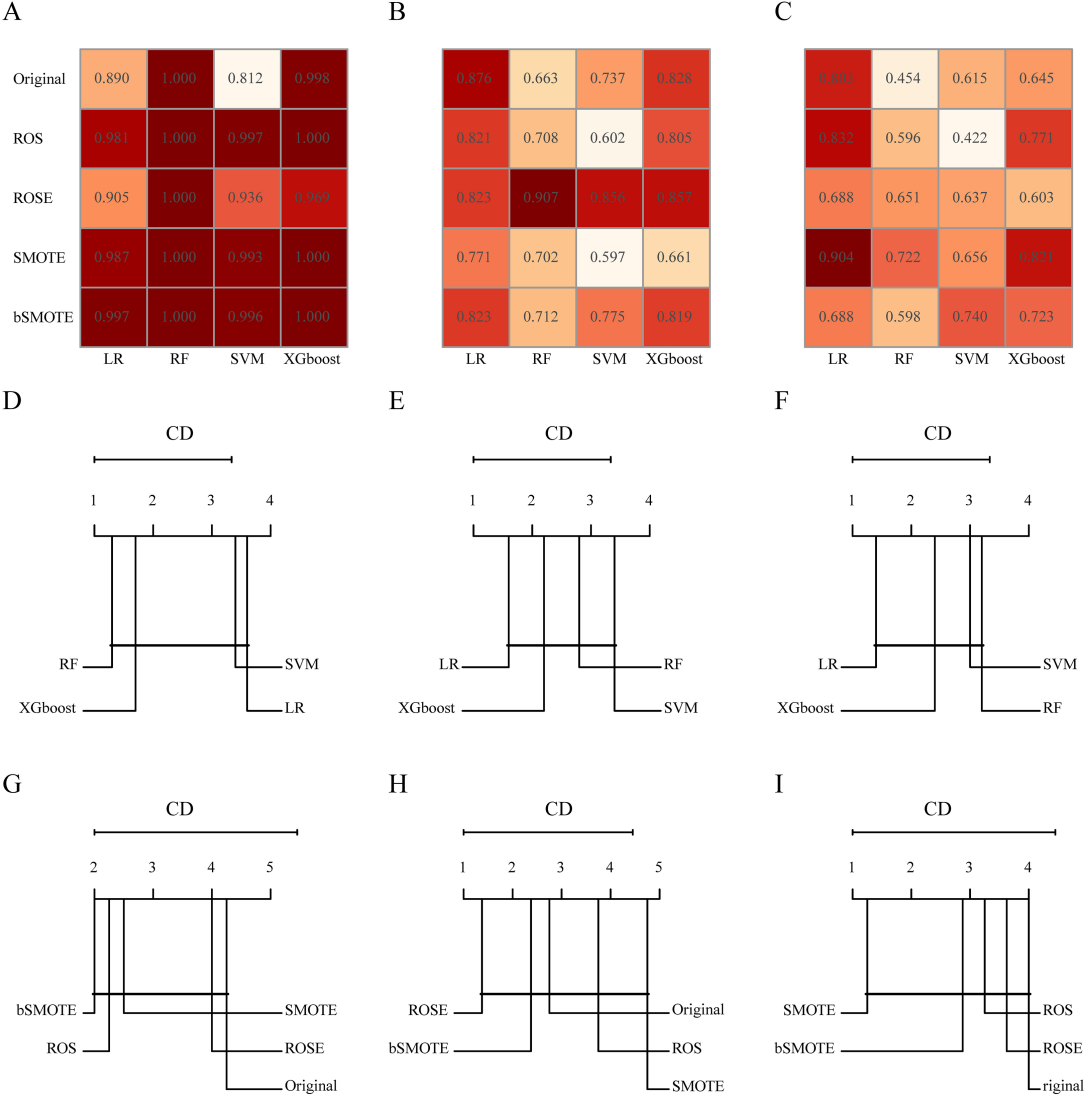
The area under the receiver operating characteristic (AUC) of different combinations of resampling techniques and machine learning algorithms in the training **(A)**, testing **(B)**, and validation **(C)** cohorts. Critical difference (CD) plots of the performance rankings over different machine learning algorithms by the *post hoc* Nemenyi test in terms of AUC in the training **(D)**, testing **(E)**, and validation **(F)** cohorts. CD plots of the performance rankings over different resampling techniques by the *post hoc* Nemenyi test in terms of AUC in the training **(G)**, testing **(H)**, and validation **(I)** cohorts. In CD plots, lower ranks correspond to better model performance, and the black bar represents the lack of statistical differences between machine learning algorithms or resampling techniques.

In addition, we constructed 20 different clinical + handcrafted + DL models using various combinations of resampling techniques and machine learning algorithms. The *post hoc* Nemenyi test was employed to compare the predictive performance of these models. The results showed that in the training cohort, the RF algorithm significantly outperformed the LR algorithm in terms of G-mean and F-measure, but not in terms of AUC. However, improvements in AUC, G-mean, or F-measure resulting from resampling techniques and machine learning algorithms were not statistically significant in either the testing or validation cohorts. CD plots were used to visualize the differences in AUC, G-mean, and F-measure (**Figure 3**, **Supplementary Figure S5**, **S6**).

## 4 Discussion

In this study, we constructed a DL radiomics model through DL and radiomics analysis based on preoperative CT images of EAT and validated its ability to identify high-risk lung cancer patients with POAF following lung lobectomy. Compared to the clinical model, the predictive performance of the DL radiomics model, which integrates clinical features and handcrafted and DL radiomics signatures, was significantly improved. Furthermore, resampling techniques and machine learning algorithms did not significantly improve model performance.

The incidence of POAF in center 1 was 3.4%, lower than that reported in previous studies (1–4), which may be attributed to the implementation of enhanced recovery after surgery protocols (22) and a lower proportion of elderly patients. However, in the low-volume center, the incidence of POAF was 8.5%, significantly higher than that in center 1, demonstrating the good transportability of the DL radiomics model. In this study, we identified advanced age (*P*=0.014) and male sex (*P*=0.030) as independent risk factors. In the three cohorts, the AUC values of the clinical model were 0.737, 0.716, and 0.630, respectively, consistent with the results of previous studies (3, 4, 10). Furthermore, the Passman score, CHADS_2_ score, and CHA_2_DS_2_-VASc score have been reported to identify high-risk patients with POAF (23–26). However, no significant differences were observed between patients with and without POAF in the three cohorts.

Artificial intelligence, particularly radiomics and deep learning, has rapidly emerged as a transformative field in translational oncology, serving as a critical bridge between medical imaging and precision medicine (27–29). In the context of lung cancer, radiomics has demonstrated broad application prospects in diagnosis, treatment response evaluation, and prognosis prediction (30–32). In recent years, the region of interest in radiomics has extended beyond the tumor itself. Radiomics features of EAT have been shown to effectively predict atrial fibrillation, mortality in acute pulmonary embolism, and myocardial ischemia (33–35). Our previous studies demonstrated that the handcrafted radiomics signatures of EAT achieved acceptable performance in predicting atrial fibrillation after pulmonary endarterectomy and coronary artery bypass grafting (16, 17). Therefore, radiomics analysis of EAT shows potential in identifying POAF in patients undergoing lung lobectomy.

DL radiomics features, particularly 3D DL features, can improve the performance of predictive models (18, 19). However, no relevant studies have been conducted on the DL radiomics analysis of EAT. In our study, we aimed to investigate the incremental value of handcrafted and DL radiomics signatures in POAF prediction. We extracted 1,130 handcrafted radiomics features and 512 DL radiomics features from each VOI, ultimately constructing handcrafted DL radiomics signatures after feature selection. The results of the Delong test and NRI analysis demonstrated that the DL radiomics model, integrating clinical features and handcrafted and DL radiomics signatures, performed satisfactorily in all cohorts, with significantly better predictive ability than the clinical model. However, the biological interpretation of radiomic features remains largely unclear, which limits a deeper understanding of their predictive mechanism and potential clinical translation. A relevant study by Mancio et al. in patients undergoing aortic valve replacement showed that a radiomics model combining EAT features and volume effectively discriminated preoperative POAF patients from those in sinus rhythm, achieving an AUC of 0.80 (95% CI: 0.68–0.92). Furthermore, proteomic analysis in that study revealed that POAF was associated with upregulation of inflammatory and prothrombotic proteins, along with downregulation of cardioprotective proteins with anti-inflammatory and antilipotoxic functions (15). Unfortunately, due to the retrospective nature of our study, we were unable to perform biologically validated assays (e.g., proteomics or histology) to explore the molecular correlates of the EAT radiomic features identified here. In our previous study, EAT segmentation was performed manually, which was time-consuming and labor-intensive. In this study, we employed the TIMESlice software to automatically segment EAT, significantly reducing the segmentation time and facilitating its clinical application.

Currently, imbalanced data remain a significant challenge for prediction models, often leading to prediction misclassification (36). Previous studies have demonstrated that resampling techniques and machine learning algorithms are effective methods for addressing the imbalance problem (37–39). In this study, we adopted the classical linear classification model constructed by the LR algorithm as the baseline model. Additionally, machine learning algorithms such as RF, SVM, and XGBoost, which have been reported to effectively address imbalance problems, were also selected (38, 40). Furthermore, considering the small number of patients with POAF, we employed four oversampling methods to balance the distribution of patients and ultimately constructed 20 different combination models. Currently, most studies only compare numerical values, lacking comprehensive statistical analysis. In our study, we employed the Friedman test and *post hoc* Nemenyi test to compare the predictive performance of different combinations of resampling techniques and machine learning algorithms, with results visualized using CD plots. The results indicated that neither machine learning algorithms nor resampling techniques significantly improved model performance in terms of AUC. G-mean and F-measure are useful evaluation metrics for imbalanced datasets. Unfortunately, we did not observe significant improvements in terms of G-mean and F-measure with machine learning algorithms and resampling techniques, contrary to previous studies (37). However, the study by Hubert S. Gabryś et al. found no advantage of resampling techniques in improving model performance (41). Due to the specific clinical challenges and the intrinsic characteristics of the data, there is no consensus on the optimal combination of machine learning algorithms and resampling techniques. Further research is required to comprehensively compare the performance of various resampling methods and machine learning algorithms.

This study has several limitations. First, due to the retrospective study design, some potential clinical features associated with POAF were not included, which may have limited the diagnostic performance. Second, it is important to note that the data for this study were retrospectively collected from two centers within a single healthcare network. While this provided internal consistency in imaging protocols, it may limit the generalizability of the findings. Furthermore, the sample size of the external validation cohort was limited, and future large-scale, multi-institutional prospective studies involving external validation cohorts from independent networks are essential to confirm the broad applicability of our model. Third, the relatively low incidence of POAF in our cohort may constrain the statistical power of the analysis and heighten the risk of model overfitting. While we employed resampling methods applied to alleviate class imbalance, further investigation into advanced feature selection and systematic hyperparameter tuning may offer valuable pathways for enhancing model generalizability and performance. Finally, the biological significance of radiomics features, particularly DL features, requires further investigation to enhance understanding and clinical application. The utilization of gradient-weighted class activation maps and perturbation-based explainable artificial intelligence techniques and the integration of radiomic features with other interpretable omics data, such as tissue metabolomics, all represent promising potential avenues for enhancing model interpretability (42–44).

## 5 Conclusion

In summary, the DL radiomics model based on preoperative EAT images effectively identified high-risk lung cancer patients with POAF following lung lobectomy and offers a novel tool for risk stratification. Neither machine learning algorithms nor resampling techniques significantly improved model performance in terms of AUC, G-mean, and F-measure.

## Data Availability

The raw data supporting the conclusions of this article will be made available by the authors, without undue reservation.
