# Supplementary material for: Deep learning radiomics model of epicardial adipose tissue for predicting postoperative atrial fibrillation after lung lobectomy in lung cancer patients
Source: Front Oncol. 2025 Oct 13;15:1623248. doi: 10.3389/fonc.2025.1623248 (PMC12554601; doi:10.3389/fonc.2025.1623248)
Supplement: Supplementary file 1 [file DataSheet1.docx]

Supplementary Material

# Supplementary Figures


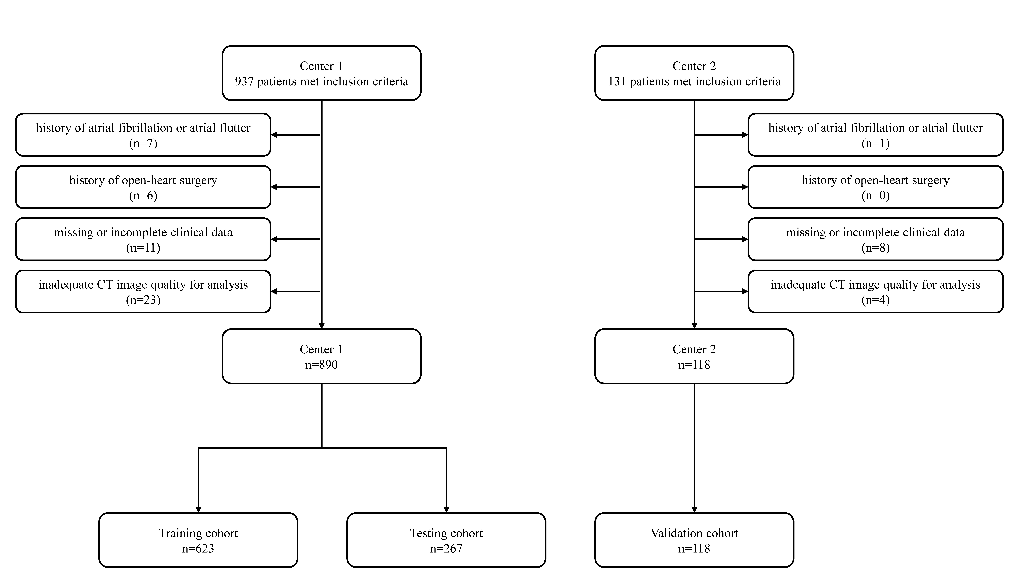

**Supplementary Figure S1**. The workflow of this study.


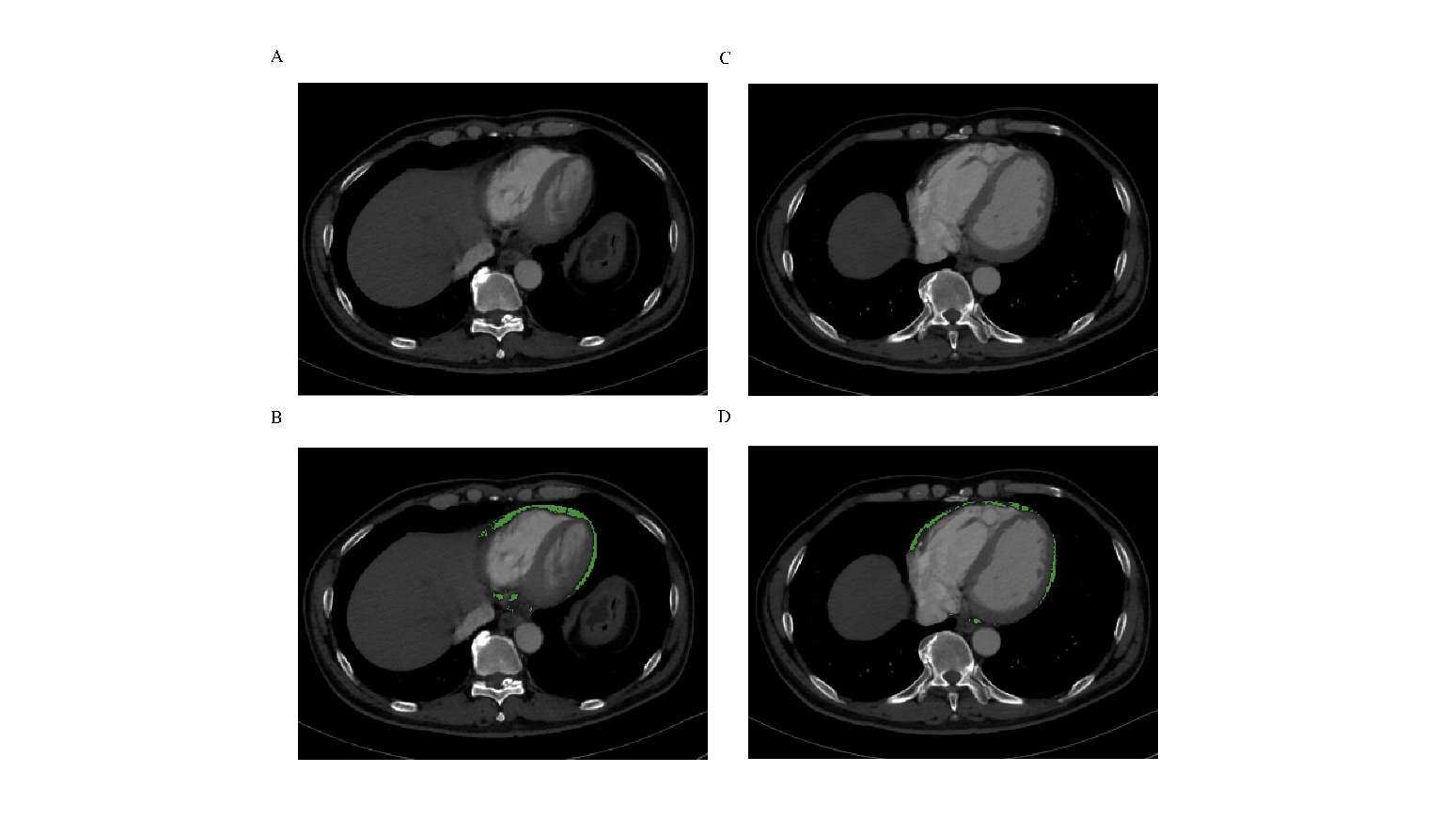


Supplement Figure S2. Original contrast-enhanced chest CT images (A, C). The green areas in the image indicate the epicardial adipose tissue (B, D).

**Supplementary Figure S3**. The distribution of postoperative atrial fibrillation (POAF) and non-POAF patients before and after applying different re-sampling techniques. The proportions of POAF and non-POAF patients in the five datasets were 21:602, 609:646, 598:602, 299:324, and 597:602, respectively.

ROS, random oversampling; ROSE, random over-sampling examples; SMOTE, synthetic minority oversampling technique; bSMOTE, Borderline-SMOTE.


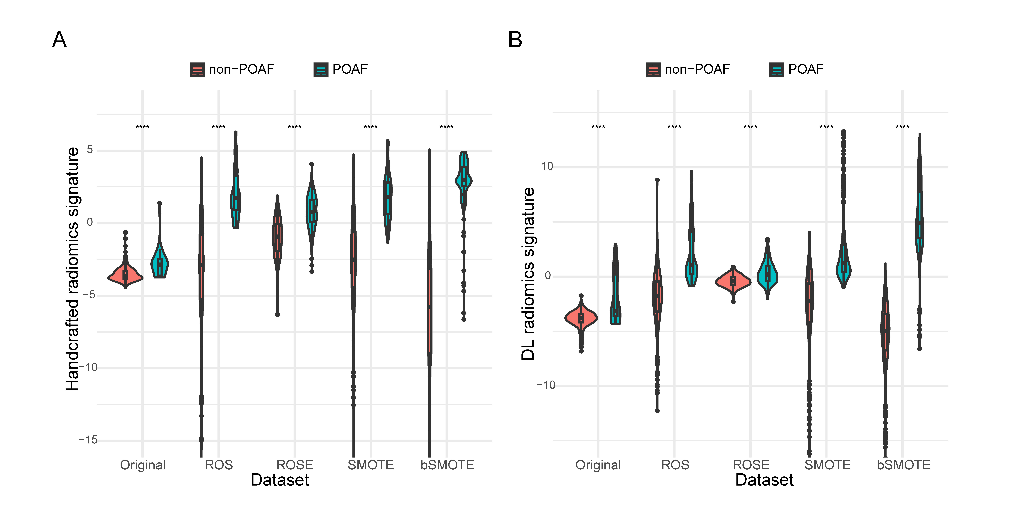


**Supplementary Figure S4.** Violin plot of the distribution of handcrafted radiomics signatures (A) and deep learning radiomics signatures (B) in the original and resampling datasets.

POAF, postoperative atrial fibrillation.


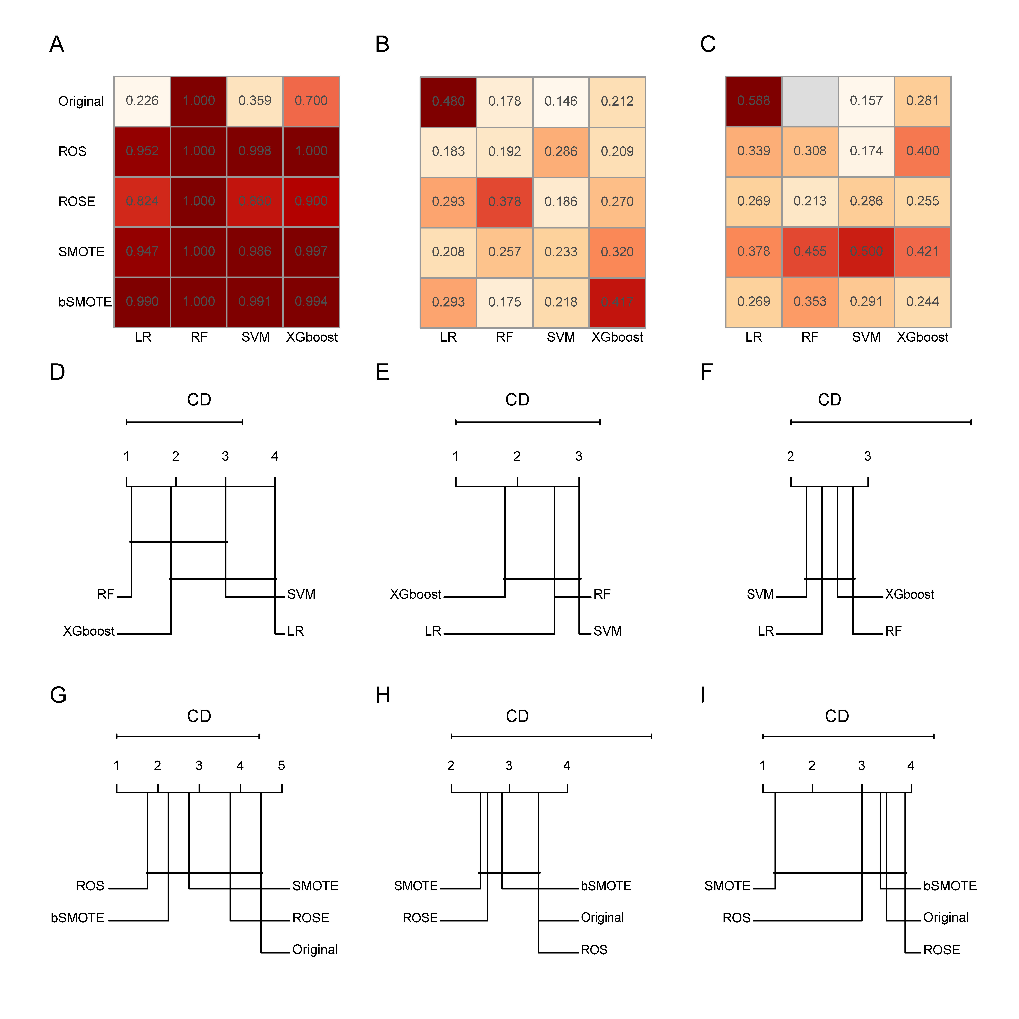


**Supplementary Figure S5.** The G-means of different combinations of re-sampling techniques and machine learning algorithms in the training (A), testing (B), and validation(C) cohorts. Critical distance (CD) plots of the performance rankings over different machine learning algorithms by post hoc Nemenyi test in terms of G-mean in the training (D), testing (E), and validation (F) cohorts. CD plots of the performance rankings over different re-sampling techniques by post hoc Nemenyi test in terms of G-mean in the training (G), testing (H), and validation (I) cohorts.

ROS, random oversampling; ROSE, random over-sampling examples; SMOTE, synthetic minority oversampling technique; bSMOTE, Borderline-SMOTE; SVM, support vector machine; LR, logistic regression; RF, random forest


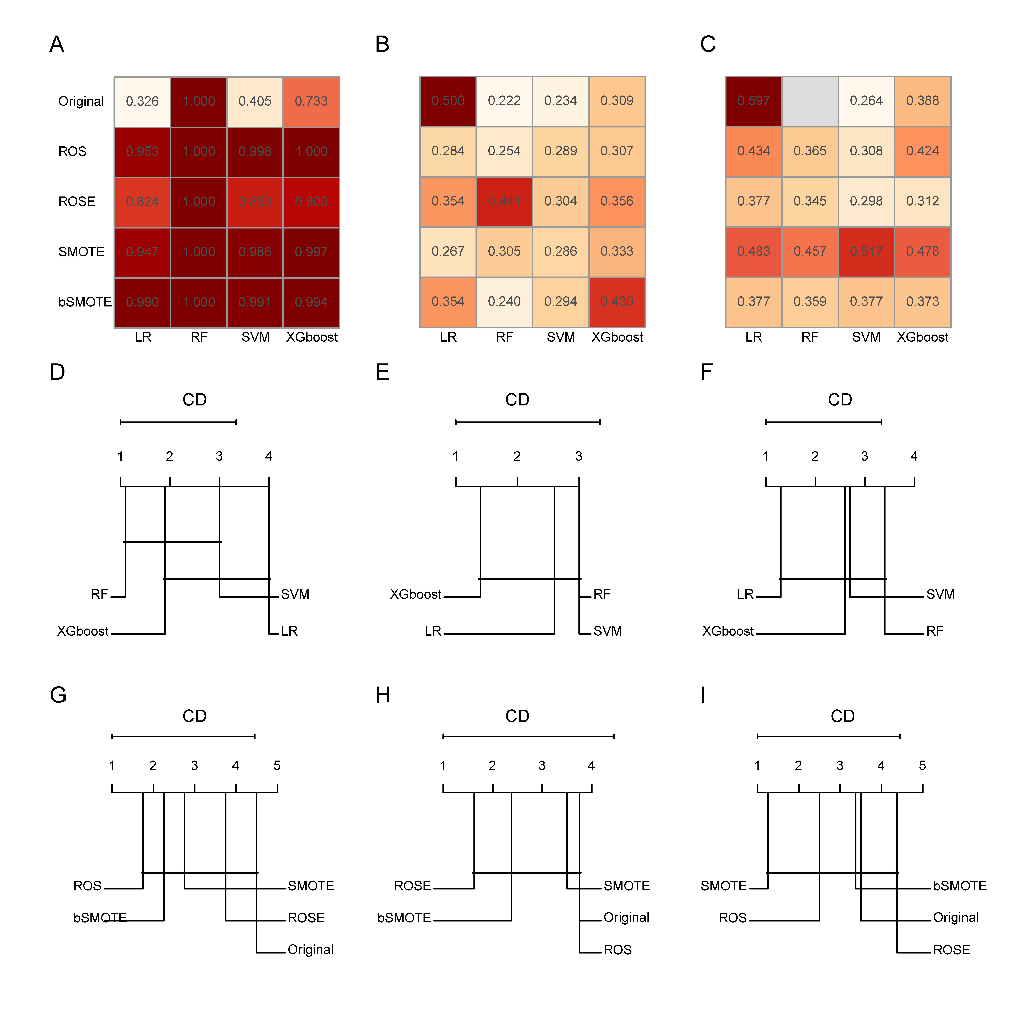


**Supplementary Figure S6**. The F-measures of different combinations of re-sampling techniques and machine learning algorithms in the training (A), testing (B), and validation(C) cohorts. Critical distance (CD) plots of the performance rankings over different machine learning algorithms by post hoc Nemenyi test in terms of F-measure in the training (D), testing (E), and validation (F) cohorts. CD plots of the performance rankings over different re-sampling techniques by post hoc Nemenyi test in terms of F-measure in the training (G), testing (H), and validation (I) cohorts.

ROS, random oversampling; ROSE, random over-sampling examples; SMOTE, synthetic minority oversampling technique; bSMOTE, Borderline-SMOTE; SVM, support vector machine; LR, logistic regression; RF, random forest

# Supplementary Tables

**Supplementary Table S1. Imaging Equipment and Acquisition Parameters.**

| CT scanners | slice thickness | tube voltage | tube current | pitch | reconstruction layer thickness |
| --- | --- | --- | --- | --- | --- |
| Center 1 |  |  |  |  |  |
| Philips Brilliance iCT | 5mm | 120KV | automatic | 1.14 | 1mm |
| GE Revolution CT | 5mm | 120KV | SMART | 0.992 | 1.25mm |
| SIEMENS Somatom go.FitCT | 5mm | 120KV | automatic | 1 | 1mm |
| United Imaging uCT960 | 5mm | 120KV | automatic | 0.9688 | 1mm |
| Center 2 | e |  |  |  |  |
| GE Revolution CT | 5mm | 120KV | SMART | 0.992 | 1.25mm |
| SIEMENS Somatom go.FitCT | 5mm | 120KV | automatic | 1 | 1mm |

**Supplementary Table S2.** Clinical features between patients in the training and testing cohorts.

| Features | Training cohort  n = 623 | Testing cohort  n = 267 | *p*-value |
| --- | --- | --- | --- |
| POAF | 21 (3.4) | 9 (3.4) | 1.000 |
| Sex |  |  | 0.849 |
| Female | 289 (46.4) | 122 (45.7) |  |
| Male | 334 (53.6) | 145 (54.3) |  |
| Age (year) | 62 (56, 69) | 61 (55, 68) | 0.418 |
| BMI (kg/m2) | 24.2 (22.0, 26.7) | 24.2 (22.0, 27.0) | 0.545 |
| Hypertension | 193 (31.0) | 71 (26.6) | 0.189 |
| DM | 69 (11.1) | 30 (11.2) | 0.944 |
| CAD | 54 (8.7) | 22 (8.2) | 0.834 |
| CVD | 46 (7.4) | 18 (6.7) | 0.734 |
| PAD | 16 (2.6) | 4 (1.5) | 0.324 |
| Smoking history | 223 (35.8) | 101 (37.8) | 0.563 |
| Heart rate (bpm) | 68 (61, 76) | 68 (61, 75) | 0.941 |
| WBC (10^9^/L) | 5.55 (4.50, 6.73) | 5.47 (4.54, 7.00) | 0.425 |
| Neutrophil (10^9^/L) | 2.44 (3.26, 4.18) | 3.25 (2.52, 4.33) | 0.411 |
| Lymphocytes (10^9^/L) | 1.64 (1.32, 1.98) | 1.68 (1.30, 2.03) | 0.674 |
| Platelets (10^9^/L) | 230 (195, 276) | 240 (194, 275) | 0.417 |
| CCB | 82 (13.2) | 34 (12.7) | 0.862 |
| Metoprolol | 26 (4.2) | 8 (3.0) | 0.401 |
| Neoadjuvant therapy | 86 (13.8) | 44 (16.5) | 0.300 |
| CHADS2 score ≥ 2 | 88 (14.1) | 35 (13.1) | 0.687 |
| CHA2DS2-VASc score ≥ 5 | 18 (2.9) | 5 (1.9) | 0.381 |
| Passman score ≥ 4 | 206 (33.1) | 74 (27.7) | 0.115 |

Categorical variables shown with frequency and percentage; continuous variables shown with median and interquartile range; POAF, postoperative atrial fibrillation; BMI, body mass index; DM, diabetes mellitus; CAD, coronary heart disease; CVD, cerebrovascular disease; PAD, peripheral arterial disease; bpm, beat per minute; WBC, white blood cell; CCB, calcium channel blocker.

**Supplementary Table S3.** Clinical features between patients in the training and validation cohorts.

| Features | Training cohort  n = 623 | Validation cohort  n = 118 | *p*-value |
| --- | --- | --- | --- |
| POAF | 21 (3.4) | 10 (8.5) | 0.022 |
| Sex |  |  | 0.422 |
| Female | 289 (46.4) | 50 (42.4) |  |
| Male | 334 (53.6) | 68 (57.6) |  |
| Age (year) | 62 (56, 69) | 66 (59, 72) | 0.001 |
| BMI (kg/m2) | 24.2 (22.0, 26.7) | 23.5 (21.6, 25.7) | 0.037 |
| Hypertension | 193 (31.0) | 32 (27.1) | 0.403 |
| DM | 69 (11.1) | 15 (12.7) | 0.607 |
| CAD | 54 (8.7) | 15 (12.7) | 0.166 |
| CVD | 46 (7.4) | 8 (6.8) | 0.817 |
| PAD | 16 (2.6) | 2 (1.7) | 0.572 |
| Smoking history | 223 (35.8) | 46 (39.0) | 0.509 |
| Heart rate (bpm) | 68 (61, 76) | 76 (68, 84) | <0.001 |
| WBC (10^9^/L) | 5.55 (4.50, 6.73) | 5.79 (4.89, 7.32) | 0.037 |
| Neutrophil (10^9^/L) | 2.44 (3.26, 4.18) | 3.72 (2.96, 4.97) | 0.001 |
| Lymphocytes (10^9^/L) | 1.64 (1.32, 1.98) | 1.60 (1.19, 2.02) | 0.302 |
| Platelets (10^9^/L) | 230 (195, 276) | 241 (194, 296) | 0.118 |
| CCB | 82 (13.2) | 19 (16.1) | 0.393 |
| Metoprolol | 26 (4.2) | 9 (7.6) | 0.105 |
| Neoadjuvant therapy | 86 (13.8) | 3 (2.5) | 0.001 |
| CHADS2 score ≥ 2 | 88 (14.1) | 15 (12.7) | 0.684 |
| CHA2DS2-VASc score ≥ 5 | 18 (2.9) | 5 (4.2) | 0.628 |
| Passman score ≥ 4 | 206 (33.1) | 78 (66.1) | <0.001 |

Categorical variables shown with frequency and percentage; continuous variables shown with median and interquartile range; POAF, postoperative atrial fibrillation; BMI, body mass index; DM, diabetes mellitus; CAD, coronary heart disease; CVD, cerebrovascular disease; PAD, peripheral arterial disease; bpm, beat per minute; WBC, white blood cell; CCB, calcium channel blocker.

**Supplementary Table S4.** Clinical features between patients with and without POAF in the testing cohort.

| Features | Non-POAF  n = 258 | POAF  n = 9 | *p*-value |
| --- | --- | --- | --- |
| Sex |  |  | 0.187 |
| Female | 120 (46.5) | 2 (22.2) |  |
| Male | 138 (53.5) | 7 (77.8) |  |
| Age (year) | 61 (55, 68) | 67 (60, 70) | 0.090 |
| BMI (kg/m^2^) | 24.2 (22.0, 27.0) | 23.0 (21.4, 27.4) | 0.659 |
| Hypertension | 66 (25.6) | 5 (26.6) | 0.106 |
| DM | 29 (11.2) | 1 (11.1) | 1.000 |
| CAD | 21 (8.1) | 1 (11.1) | 1.000 |
| CVD | 16 (6.2) | 2 (22.2) | 0.227 |
| PAD | 4 (1.6) | 0 (0) | 1.000 |
| Smoking history | 96 (37.2) | 5 (55.6) | 0.444 |
| Heart rate (bpm) | 68 (61, 75) | 72 (63, 83) | 0.257 |
| WBC (10^9^/L) | 5.46 (4.50, 6.96) | 6 .00 (5.16, 8.50) | 0.126 |
| Neutrophil (10^9^/L) | 3.22 (2.49, 4.28) | 4.11 (2.88, 5.60) | 0.093 |
| Lymphocytes (10^9^/L) | 1.68 (1.30, 2.03) | 1.51 (1.25, 1.96) | 0.520 |
| Platelets (10^9^/L) | 240 (167, 276) | 217 (164, 282) | 0.386 |
| CCB | 31 (12.0) | 3 (33.3) | 0.168 |
| Metoprolol | 8 (3.1) | 0 (0) | 1.000 |
| Neoadjuvant therapy | 43 (16.7) | 1 (11.1) | 1.000 |
| CHADS_2_ score ≥ 2 | 33 (12.8) | 2 (22.2) | 0.748 |
| CHA_2_DS_2_-VASc score ≥ 5 | 4 (1.6) | 1 (11.1) | 0.159 |
| Passman score ≥ 4 | 69 (26.7) | 5 (55.6) | 0.129 |

Categorical variables shown with frequency and percentage; continuous variables shown with median and interquartile range; POAF, postoperative atrial fibrillation; BMI, body mass index; DM, diabetes mellitus; CAD, coronary heart disease; CVD, cerebrovascular disease; PAD, peripheral arterial disease; bpm, beat per minute; WBC, white blood cell; CCB, calcium channel blocker.

**Supplementary Table S5.** Clinical features between patients with and without POAF in the validation cohort.

| Features | Non-POAF  n =108 | POAF  n = 10 | *p*-value |
| --- | --- | --- | --- |
| Sex |  |  | 0.861 |
| Female | 45 (41.7) | 5 (50.0) |  |
| Male | 63 (58.3) | 5 (50.0) |  |
| Age (year) | 66 (57, 71) | 71 (68, 76) | 0.009 |
| BMI (kg/m^2^) | 23.5 (21.6, 25.7) | 23.4 (21.6, 26.0) | 0.674 |
| Hypertension | 27 (25.0) | 5 (50.0) | 0.184 |
| DM | 13 (12.0) | 2 (20.0) | 0.820 |
| CAD | 12 (11.1) | 3 (30.0) | 0.223 |
| CVD | 7 (6.5) | 1 (10.0) | 1.000 |
| PAD | 2 (1.9) | 0 (0) | 1.000 |
| Smoking history | 42 (38.9) | 4 (40.0) | 1.000 |
| Heart rate (bpm) | 76 (68, 84) | 79 (72, 83) | 0.727 |
| WBC (10^9^/L) | 5.71 (4.86, 7.26) | 6.30 (5.17, 7.64) | 0.460 |
| Neutrophil (10^9^/L) | 3.69 (2.96, 4.89) | 3.90 (2.89, 5.85) | 0.660 |
| Lymphocytes (10^9^/L) | 1.61 (1.19, 2.02) | 1.44 (1.12, 2.46) | 0.820 |
| Platelets (10^9^/L) | 237 (185, 292) | 267 (234, 352) | 0.078 |
| CCB | 16 (14.8) | 3 (30.0) | 0.424 |
| Metoprolol | 7 (6.5) | 2 (20.0) | 0.359 |
| Neoadjuvant therapy | 3 (2.8) | 0 (0) | 1.000 |
| CHADS_2_ score ≥ 2 | 12 (11.1) | 3 (30.0) | 0.223 |
| CHA_2_DS_2_-VASc score ≥ 5 | 4 (3.7) | 1 (10.0) | 0.363 |
| Passman score ≥ 4 | 70 (64.8) | 8 (80.0) | 0.534 |

Categorical variables shown with frequency and percentage; continuous variables shown with median and interquartile range; POAF, postoperative atrial fibrillation; BMI, body mass index; DM, diabetes mellitus; CAD, coronary heart disease; CVD, cerebrovascular disease; PAD, peripheral arterial disease; bpm, beat per minute; WBC, white blood cell; CCB, calcium channel blocker.

Sample size estimation

The sample size was estimated using the “pmsampsize” package (1), which is recommended for sample size calculation in the development of artificial intelligence-based healthcare prediction models (2). Based on the performance observed in the training cohort (target C-statistic = 0.89, number of parameters = 4, event prevalence = 0.034), the results indicated that a minimum sample size of 399 was required for model development. Our training cohort included 623 patients, which exceeds this minimum requirement.

1. Riley RD, Ensor J, Snell KIE, Harrell FE Jr, Martin GP, Reitsma JB, et al. Calculating the sample size required for developing a clinical prediction model. BMJ. (2020) 18;368:m441. doi: 10.1136/bmj.m441

2. Riley RD, Ensor J, Snell KIE, Archer L, Whittle R, Dhiman P, et al. Importance of sample size on the quality and utility of AI-based prediction models for healthcare. Lancet Digit Health. (2025) 7(6):100857. doi: 10.1016/j.landig.2025.01.013
